# Up-Down-Like Background Spiking Can Enhance Neural Information Transmission

**DOI:** 10.1523/ENEURO.0282-17.2017

**Published:** 2018-01-18

**Authors:** Felix Droste, Benjamin Lindner

**Affiliations:** 1 Bernstein Center for Computational Neuroscience, Berlin 10115, Germany; 2 Department of Physics, Humboldt Universität zu Berlin, Berlin 12489, Germany

**Keywords:** information transmission, modelling, network, spontaneous activity, up-down states

## Abstract

How neurons transmit information about sensory or internal signals is strongly influenced by ongoing internal activity. Depending on brain state, this background spiking can occur asynchronously or clustered in up states, periods of collective firing that are interspersed by silent down states. Here, we study which effect such up-down (UD) transitions have on signal transmission. In a simple model, we obtain numerical and analytical results for information theoretic measures. We find that, surprisingly, an UD background can benefit information transmission: when background activity is sparse, it is advantageous to distribute spikes into up states rather than uniformly in time. We reproduce the same effect in a more realistic recurrent network and show that signal transmission is further improved by incorporating that up states propagate across cortex as traveling waves. We propose that traveling UD activity might represent a compromise between reducing metabolic strain and maintaining information transmission capabilities.

## Significance Statement

Spontaneous background activity in the cortex shapes the information transmission between neural populations. While asynchronous-irregular (AI) spontaneous activity has received lots of attention from theoreticians, the important case where the background switches between levels of high and low activity (up and down states) is still poorly understood. Here, we put forward a theoretical framework for computing the information transmission in the presence of up-down (UD) transitions. We show that an UD background can benefit information transmission when the firing rate in the background is low, indicating that such regimes may be well suited to maintain basic information transmission capabilities at lowered firing rates.

## Introduction

The behavioral state of an animal is reflected in the activity of neural populations, the so-called brain state. Roughly, two extremes of a continuum of brain states have been distinguished (Harris and Thiele, 2011): an asynchronous-irregular (AI) or desynchronized regime (Renart et al., 2010), in which neurons fire independently at an approximately constant rate, and an up-down (UD) or synchronized regime, where the firing rate jumps between a high and a low level, termed up and down states (Steriade et al., 1993; Cowan and Wilson, 1994). The AI regime is typically observed during attentive, task-related behavior, while UD switching is traditionally associated with anesthesia or slow-wave sleep (Steriade et al., 2001), but has more recently also been observed during quiet wakefulness (Petersen et al., 2003; Luczak et al., 2007; Poulet et al., 2012; Luczak et al., 2013).

Neural information processing involves the communication between populations of neurons (Gerstner et al., 2014). For a signal transmitted by one population, the spontaneous activity of other populations constitutes a background (Chance et al., 2002; Destexhe and Contreras, 2006), the nature of which depends on brain state. Experiments studying the influence of background activity have focused on the response to sensory signals, which is strongly influenced by ongoing activity (Arieli et al., 1996) and top-down input from higher cortical regions (Gilbert and Sigman, 2007). It seems likely that non-sensory internal signals are equally influenced by background populations, although disentangling signal and background experimentally is, of course, difficult in this case.

How signal transmission is influenced by an AI background has been thoroughly studied (Brunel et al., 2001; Lindner and Schimansky-Geier, 2001; Chance et al., 2002; Larkum et al., 2004; Vogels and Abbott, 2005; Kumar et al., 2008), but in theoretical work the case of a UD background has largely been neglected. For one, sensory signals may face such a background during quiet wakefulness. A growing number of experimental studies have therefore investigated the effect that a UD regime has on the transmission of time-dependent sensory stimuli (Goard and Dan, 2009; Marguet and Harris, 2011; Luczak et al., 2013; Zagha et al., 2013; Pachitariu et al., 2015) and, most recently, UD-like dynamics has been even linked to selective attention (Engel et al., 2016). But also in the case of UD activity during slow-wave sleep, it is important to understand consequences for information transmission. Here, internal signals, mediating, for instance, the consolidation of memories (Diekelmann and Born, 2010), need to be transmitted. This calls for an extension of the theoretical framework that has been successfully used to study AI backgrounds to the UD case.

A better theoretical understanding of information transmission under UD regimes may also help to elucidate their functional role, which is still debated. Closely linked is the question about the purpose of sleep, for which various hypotheses exist (Watson and Buzsáki, 2015), the most prominent one of which is certainly memory consolidation (Maquet, 2001; Diekelmann and Born, 2010; Rasch and Born, 2013). Of interest for our work is that the UD regime in slow-wave sleep has been hypothesized to allow synaptic homeostasis (Tononi and Cirelli, 2006) and to enable regenerative cellular mechanisms that cannot function under the strain of higher overall firing rates (Vyazovskiy and Harris, 2013). This last hypothesis raises the question why a lower firing rate is realized by introducing pauses, the down states, rather than in a way that maintains an AI regime. In this theoretical study, we propose that the answer may lie in the respective signal transmission properties. We compare how a time-dependent signal is transmitted by a neural population that is subject to either a UD or an AI background and show that a UD regime may be preferable when background rates are low, because, for a given budget of background spikes, it allows more information to be transmitted. In other words, if overall background rates are lowered, the optimal kind of background switches from an AI spiking to a UD regime and that is why the latter regime is indeed observed in brain states with low overall firing rate.

## Materials and Methods

We first consider a simplified setup, in which the neurons of the readout population are uncoupled, the signal enters as a current, and UD durations are exponentially distributed. Then, we describe its extension to a recurrent network with rate-coded signal and gamma-distributed UD durations, as well as a simple way to incorporate traveling UD states. The parameters used in all model variants are summarized in Table 1.

**Table 1.** Parameters used if not indicated otherwise

Uncoupled population		
*N*	1000	Size of readout population
*τ*	20 ms	Membrane time constant
*V*_0_	15 mV	Resting potential
$\tau_{\text{ref}}$	1 ms	Refractory period
*J*_ext_	0.1 mV	Mean weight of background spikes
$v_{\text{T}}$	20 mV	Threshold voltage
$v_{\text{R}}$	10 mV	Reset voltage
*N*_B_	1000	Number of background neurons
${\bar{r}}_{\text{B}}$	1.35 Hz	Mean background rate
$\tau_{\text{U}}$	333 ms	Mean up state duration
$\tau_{\text{D}}$	200 ms	Mean down state duration
α	1	*γ* Distribution scale parameter
ϵ	0.3 mV	Signal standard deviation
*f*_0_	0 Hz	Lower signal cutoff frequency
*f*_C_	75 Hz	Upper signal cutoff frequency
Δt	0.1 ms	Simulation time step
*T*	4000 ms	Simulation time for one trial
Recurrent network (where different from above)		
*N*_S_	1000	Size of signal population
*r*_S_	1 Hz	Baseline rate of signal population
$\epsilon_{\text{S}}$	0.15	Relative rate modulation
${\bar{r}}_{\text{B}}$	1.9 Hz	Mean background rate
$\tau_{\text{U}}$	200 ms	Mean up state duration
α	50	*γ* Distribution scale parameter
*N*_E_	10,000	Size of excitatory population
*N*_I_	2500	Size of inhibitory population
*J*	0.1 mV	Weight of recurrent connections
*C*_E_	1000	Number excitatory connections
*C*_I_	250	Number inhibitory connections
*V*_0_	11 mV	Resting potential
*g*	4.5	Relative strength of inhibition
*D*	1.5 ms	Delay
Traveling UD		
*l*	4 mm	Extent of background population
*c*	10 mm/s	Propagation speed

### Readout neuron dynamics in the uncoupled case

The readout population consists of *N* = 1000 leaky integrate-and-fire (LIF) neurons. Their dynamics between spikes is given by

(1) $$\tau {\overset{˙}{v}}_{n}=V_{0}-v_{n}+\epsilon s(t)+\tau \underset{i}{\sum }J_{\text{ext},n,i}\delta (t-t_{n,i}^{*}),$$

where *τ* = 20 ms is the membrane time constant, *V*_0_ the resting potential, ϵs(t) the input signal, $\left\{t_{n,i}^{*}\right\}$ the set of arrival times of all background spikes to the *n*th neuron, and $J_{\text{ext},n,i}$ the weight of the *i*th background spike. Equation 1 is supplemented by a fire-and-reset rule: whenever $v=v_{\text{T}}=20$ mV, a spike is registered; mathematically, this is represented as a *δ*-peak in the neuron’s spike train *x_n_*(*t*). The voltage is then reset to $v_{\text{R}}=10$ mV where it is clamped for an absolute refractory period $\tau_{\text{ref}}=1$ ms.

### Background input

The weights of background spikes, $J_{\text{ext},n,i}$, are drawn from an exponential distribution with mean *J*_ext._ Such a skewed distribution is a more realistic assumption than identical spike weights; however, our primary reason for using this is the analytical tractability it brings along (Richardson and Swarbrick, 2010; Droste and Lindner, 2017a). Background spikes occur according to an inhomogeneous Poisson process with rate $\left\langle \sum_{i}\delta (t-t_{n,i}^{*})\right\rangle =N_{\text{B}}r_{\text{B}}(t)$. Here, *N*_B_ is the number of background neurons from which each readout neuron receives inputs and

(2) $$r_{\text{B}}(t):=\{\begin{array}{ll} {\overset{¯}{r}}_{\text{B}} & \text{in}\;\text{AI}\;\text{regime}\\ \eta (t) & \text{in}\;\text{UD}\;\text{regime} \end{array},$$

where *η*(*t*) is a Markovian dichotomous process that jumps between up states ($\eta (t)={\bar{r}}_{\text{B}}\;\cdot \;(\tau_{\text{U}}+\tau_{\text{D}})/\tau_{\text{U}}$) and down states (*η*(*t*) = 0) at constant rates $k_{+}=1/\tau_{\text{U}}$ (up to down) and $k_{-}=1/\tau_{\text{D}}$ (down to up).

### Signal

The signal *s*(*t*) is band-limited Gaussian noise with a flat spectrum given by $S_{ss}(f)=1/(2[f_{\text{C}}-f_{0}])[\Theta (f-f_{0})-\Theta (f-f_{\text{C}})]$. This choice implies that *s*(*t*) has unit variance; the variance of ϵs(t) is then $\epsilon^{2}$. In simulations, signals are generated by randomly drawing frequency components, consistent with $S_{ss}(f)$, which are then transformed back to the time domain.

### Output

The output of the readout population which we consider to assess information transmission is its population activity, i.e., the normalized sum over the neurons’ spike trains, *x_n_*(*t*),

(3) $$a(t)=\frac{1}{N}\sum_{n}^{N}x_{n}(t).$$

In simulations, we measure a binned version of *a*(*t*) with bin width ΔT=4 ms.

### Extension to a recurrent network with rate-coded signal and more regular UD switching

We now consider an excitatory (E) and an inhibitory (I) population. Each neuron has a fixed number of *C_E_* = 1000 (*C_I_* = 250) randomly chosen presynaptic partners from the E (I) population (autapses are excluded). The neural dynamics are now governed by

(4) $$\tau {\overset{˙}{v}}_{P,n}=V_{0}-v_{P,n}+\tau [\underset{i}{\sum }J_{\text{ext},n,i}\delta (t-t_{n,i}^{*})+J\left(\overset{C_{E}}{\underset{k}{\sum }}x_{n,k}(t-D)-g\overset{C_{I}}{\underset{l}{\sum }}x_{n,l}(t-D)\right)],$$

where P={E,I}. Here, *J* = 0.1 mV is the weight of recurrent connections, *D* = 1.5 ms is the transmission delay and *x_n_*_,_*_k_*(*t*) (*x_n_*_,_*_l_*(*t*)) is the spike train of the *n*th neuron’s *k*th (*l*th) excitatory (inhibitory) presynaptic partner. The Poisson input now also comprises spikes from the signal population,

(5) $$\left\langle \underset{i}{\sum }\delta (t-t_{n,i}^{*})\right\rangle =r_{\text{B}}(t)+N_{\text{S}}r_{\text{S}}(1+\epsilon_{\text{S}}s(t)).$$

The two-state process η(t) is no longer Markovian; instead, residence times are now distributed according to the *γ* distribution,

(6) $$p_{\text{U/D}}(T)=\frac{1}{\Gamma (\alpha ){\hat{\tau }}_{\text{U/D}}}x^{\alpha -1}e^{-\frac{T}{{\hat{\tau }}_{\text{U/D}}}},$$

where α is the shape parameter. For α=1, this recovers the case of exponentially distributed times. With increasing α, switching becomes more regular. We chose ${\hat{\tau }}_{\text{U/D}}$ such that the mean residence time $\tau_{\text{U/D}}={\hat{\tau }}_{\text{U/D}}\;\cdot \;\alpha$ remains constant.

### Traveling waves

As a simple way of incorporating traveling waves, we assign the background population of the *n*th readout neuron a position $X_{n}$ (drawn from a uniform distribution between 0 and *l* = 4 mm). Spikes are then drawn with the rate

(7) $$r_{\text{B},n}(t)=r_{\text{B}}\left(t-\frac{X_{n}}{c}\right),$$

where *c* is the propagation speed of the wave.

### Spectral measures

We use the following convention for the Fourier transform,

(8) $$\overset{˜}{x}(f)=\overset{\infty }{\underset{-\infty }{\int }}dt\;e^{2\pi ift}x(t),$$

where the back-transform differs only by the sign in the exponent. The power spectrum/cross-spectrum of two time series *x*(*t*) and *y*(*t*) is defined as

(9) $$\delta (f-f\prime )S_{xy}(f)=\left\langle \overset{˜}{x}(f){\overset{˜}{y}}^{*}(f\prime )\right\rangle ,$$

where * denotes complex conjugation. For stationary processes, the power/cross-spectrum corresponds to the Fourier transform of the auto/cross-correlation function. In simulations, we use the Fourier transform with a finite time window *T*, ${\tilde{x}}_{T}=\int_{0}^{T}dtx(t)e^{2\pi ift}$, and approximate the spectrum as

(10) $$S_{xy}^{T}(f)=\frac{\left\langle {\overset{˜}{x}}_{T}(f){\overset{˜}{y}}_{T}^{*}(f)\right\rangle }{T}.$$

### Theoretical expressions

Using the definition of the population activity Equation 3, the coherence function, Equation 23, can be written as

(11) $$\begin{array}{ll} C_{sa}(f) & =\frac{{\left|S_{sx}(f)\right|}^{2}}{\left(\frac{1}{N}S_{xx}(f)+\frac{N(N-1)}{N^{2}}S_{x_{i}x_{j}}(f)\right)S_{ss}(f)}, \end{array}$$

where $S_{sx}(f)=S_{sa}(f)$ is the cross-spectrum between the signal and a single spike train, *S_xx_*(*f*) is the power spectrum of a single spike train and $S_{x_{i}x_{j}}(f)$ the cross-spectrum between spike trains of different neurons. For sufficiently weak signals, one can assume linear response and write $S_{sx}(f)=\chi (f)S_{ss}(f)$, where *χ*(*f*) is the susceptibility or dynamical transfer function of the neuron with respect to current modulations. For the AI case, $S_{xx}^{\text{AI}}(f)\chi_{\text{AI}}(f)$ and $\chi_{\text{AI}}(f)$ are known (Richardson and Swarbrick, 2010; Droste and Lindner, 2017a). Here, spike trains are only correlated by the signal, so that $S_{x_{i}x_{j}}^{\text{AI}}(f)=|\chi_{\text{AI}}(f){|}^{2}S_{ss}(f)$.

For the UD case (with exponentially distributed UD durations and readout neurons that are uncoupled, though still strongly correlated due to the common UD switching), we have derived novel approximations for the spectral quantities, that we state below. The basic assumption of the approximation is that the down states are long compared to the membrane time constant. For an extensive exact treatment of the case of a pure telegraph noise (without shot-noise component), see Droste and Lindner (2017b).

Simple approximations for power spectrum and susceptibility are given by

(12) $$\chi_{\text{UD}}(f)=\frac{k_{-}}{k_{+}+k_{-}}\chi^{+}(f),$$

(13) $$\begin{array}{ll} S_{xx}^{\text{UD}}(f) & =\frac{k_{-}}{k_{+}+k_{-}}[S_{xx}^{+}(f)+\frac{2k_{+}{\overset{¯}{r}}_{\text{R}}^{+}{}^{2}}{{\left(2\pi f\right)}^{2}+{\left(k_{+}+k_{-}\right)}^{2}}]. \end{array}$$

The quantities marked by a ^+^ superscript refer to those of a LIF driven by an excitatory homogeneous Poisson process (the AI case) but at the up-state rate $r_{\text{up}}={\bar{r}}_{\text{B}}\cdot (\tau_{\text{U}}+\tau_{\text{D}})/\tau_{\text{U}}$. In essence, the susceptibility is just a scaled down version of the AI susceptibility, while the power spectrum contains an additional term that corresponds to the power spectrum of a dichotomous process that jumps between 0 and ${\bar{r}}_{\text{R}}^{+}$, where ${\bar{r}}_{\text{R}}^{+}$ is the (readout) firing rate in an up state. It can be approximated as

(14) $${\overset{¯}{r}}_{\text{R}}^{+}=2k_{+}ℜ\left[\overset{\infty }{\underset{0}{\int }}df\;\frac{\overset{˜}{R}(f)}{k_{+}+2\pi if}\right]+\frac{\overset{¯}{r}}{2},$$

where $\bar{r}$ is the stationary firing rate of an LIF driven by excitatory shot noise with exponentially distributed weights (occurring at the up-state rate $r_{\text{up}}$; Richardson and Swarbrick, 2010; Droste and Lindner, 2017a), and $\tilde{R}$ is the continuous part of the Fourier-transformed time-dependent rate of such an LIF with the initial condition *v*(*t* = 0) = *μ*, which can be expressed as

(15) $$\overset{˜}{R}(f)=\frac{F(\mu ,f)}{\frac{r_{\text{up}}-2\pi if}{r_{\text{up}}}F(v_{T},f)-e^{2\pi if\tau_{\text{ref}}}G(v_{R},f)}.$$

Here, F(v,f) and G(v,f) are given in terms of confluent hypergeometric functions,

(16) $$F(v,f):{=}_{1}F_{1}\left(-2\pi if\tau ;(r_{\text{in}}-2\pi if)\tau ;\frac{v-\mu }{a}\right),$$

(17) $$G(v,f):{=}_{1}F_{1}\left(-2\pi if\tau ;1+(r_{\text{in}}-2\pi if)\tau ;\frac{v-\mu }{a}\right).$$

Note that more elaborate approximations for $\chi_{\text{UD}}(f)$ and $S_{xx}^{\text{UD}}(f)$, which contain Equation 12 and Equation 13 as limit cases, have been derived (Droste, 2015). Here, the simple versions can be used because the frequency dependence of the coherence is dominated by the cross-spectrum.

Calculating the cross-spectrum is more complicated. Here, we need to distinguish whether all readout neurons receive the same UD process (the c→∞ limit in the traveling wave scenario) or whether they all receive independent background input (c→0). In the latter case, they are again only correlated by the signal, while in the former, we approximate the cross-spectrum as the sum of a signal-induced part and $S_{x_{i}x_{j}}^{\eta }(f)$, the cross-spectrum of two neurons driven only by Poissonian shot noise with a common two-state rate modulation. We have

(18) $$S_{x_{i}x_{j}}^{\text{UD}}(f)=\{\begin{array}{ll} \left|\chi_{\text{UD}}(f){|}^{2}S_{ss}(f\right) & c\to 0,\\ \begin{array}{ll} & S_{x_{i}x_{j}}^{\eta }(f)+\frac{k_{-}}{k_{+}+k_{-}}|\chi^{+}(f){|}^{2}S_{ss}(f) \end{array} & c\to \infty . \end{array}$$

We can express $S_{x_{i}x_{j}}^{\eta }(f)$ in terms of convolutions, involving again the Fourier transform of the time-dependent rate, $\tilde{R}(f)$ (Eq. 5),

(19) $$S_{x_{i}x_{j}}^{\eta }(f)\;=\;\frac{2k_{-}}{k_{+}+k_{-}}(2k_{+}R\left[\left(\left[\overset{˜}{R}\left({\overset{˜}{R}}^{*}*G+\frac{\overset{¯}{r}}{2}G\right)\right]*H\right)(f)\right]+\left[\frac{\overset{¯}{r}}{2}{\overset{¯}{r}}_{\text{R}}^{+}-{\overset{¯}{r}}_{\text{R}}^{+}{}^{2}\right]R[G(f)]\;+\frac{k_{+}{\overset{¯}{r}}_{\text{R}}^{+}{}^{2}}{{\left(k_{+}+k_{-}\right)}^{2}+{\left(2\pi f\right)}^{2}}),$$

where * denotes convolution and we have used the abbreviations

(20) $$G(f):=\frac{1}{k_{+}+2\pi if}$$

and

(21) $$H(f):=k_{+}\frac{\frac{1}{2}+i\frac{\pi }{k_{+}}f}{k_{+}^{2}+{\left(2\pi f\right)}^{2}}.$$

An analytical approximation for the silence fraction is obtained by exploiting that the UD process is Markovian and noting that the time that a down states needs to propagate across the population is given by *l* / *c*,

(22) $$\begin{array}{ll} & \;\mathrm{Pr}\left(\text{all}\;\text{down}\;\text{in}\;(t,t+\Delta T)\right)=\frac{k_{+}}{k_{+}+k_{-}}e^{-k_{-}\left(\frac{l}{c}+\Delta T\right)}. \end{array}$$

### Correlation measures

To quantify correlations among neurons within one trial, we average the Pearson correlation coefficient between the spike counts of two neurons in bins of length *T* = 100 ms over time and all pairs of neurons; to assess correlations across trials, we fix the signal (frozen noise stimulus) but not the UD switching and average the Pearson correlation coefficient over time and all pairs of trials.

## Results

Our basic modeling approach is motivated by the observation that UD switching often seems stochastic (Stern et al., 1997; Johnson et al., 2010; Mochol et al., 2015). Rather than picking a specific mechanism that generates such transitions dynamically (Contreras et al., 1996; Sanchez-Vives and McCormick, 2000; Compte et al., 2003; Holcman and Tsodyks, 2006; Mochol et al., 2015), we prescribe the statistical distributions of UD state durations in the background. This permits a clean comparison between the effects of UD versus AI backgrounds on signal transmission. Moreover, it allows us to derive analytical results for the statistics of information flow in the presence of UD states.

### UD transitions in the background enable better tracking of a time-dependent signal

**Figure 1.**
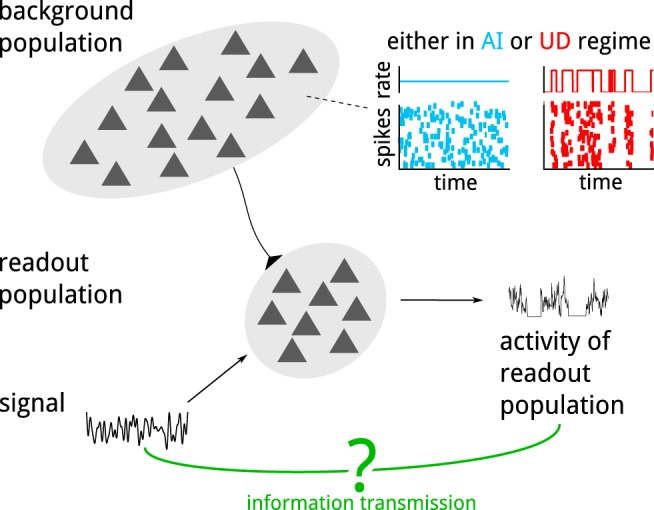
How do the transmission properties of the readout population depend on the activity of the background population? 1000 uncoupled neurons receive a common signal. We ask how the information transmission between the signal and the activity of this readout population is influenced by the dynamic regime of the background population. In particular, we compare a regime where background spikes occur uniformly in time to one where they have been redistributed into up states.

We first consider a population of *N* = 1000 uncoupled LIF neurons (the readout population), each of which receives the same signal (Fig. 1, sketch). Additionally, each readout neuron receives input from 1000 neurons that belong to the background population. These background neurons are modeled by Poisson processes that share a common time-dependent rate. We distinguish two regimes of the background population: it is either in an AI regime, in which case the rate is constant in time, or in a UD regime, in which case the rate is a stochastic process that jumps between two levels so that the time spent in each state is exponentially distributed with mean $\tau_{\text{U}}$ (up) and $\tau_{\text{D}}$ (down), respectively. We model the signal as band-limited Gaussian white noise with a cutoff frequency of *f*_C_ = 75 Hz. See the Methods section for details of this setup.

**Figure 2.**
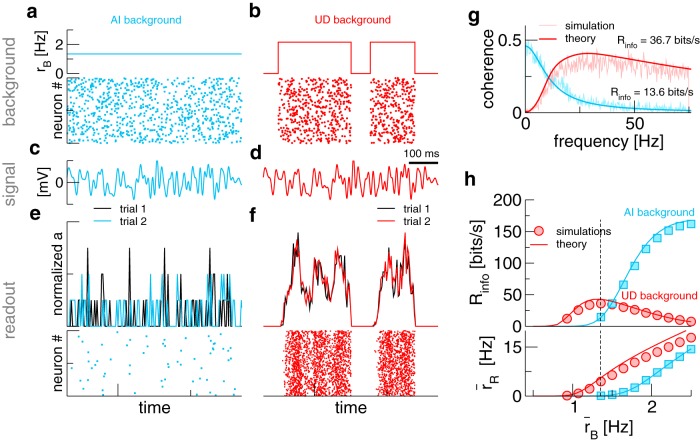
More information about the signal can be transmitted by the readout population activity *a*(*t*) if background activity undergoes transitions between up and down states. ***A***, ***B***, Firing rate of the background population and raster-plot of the spikes received by an arbitrary readout neuron. ***C***, ***D***, Signal realization (frozen noise). ***E***, ***F***, Normalized population activity a(t)/max⁡[a(t)] and spike raster of the readout population. For the population activity, two trials with the same signal (***C***, ***D***) and, in the UD case, two-state process (***B***) are shown. ***G***, Coherence between signal and activity of the readout population. ***H***, Lower bound to the mutual information rate and ${\bar{r}}_{\text{R}}$, the mean firing rate of the readout population, as a function of the mean background rate ${\bar{r}}_{\text{B}}$. The dashed line marks the ${\bar{r}}_{\text{B}}$ value used in ***A–G***. Parameters, if not indicated otherwise: ${\bar{r}}_{\text{B}}=1.35$ Hz, $\tau_{\text{U}}=333$ ms, $\tau_{\text{D}}=200$ ms.

For comparing signal transmission with an AI background and a UD background (Fig. 2*A*,*B*), we keep the mean background rate ${\bar{r}}_{B}$ fixed, i.e., over a long time window, a postsynaptic cell receives on average the same number of background spikes. In both cases, we present the same signal (Fig. 2*C*,*D*). Although the mean background rate is the same, the response of the readout population differs drastically: in the AI case (Fig. 2*E*), very few spikes are elicited. Here, the population activity *a*(*t*) seems to be little related to the signal and is not reproducible on repeated stimulation (to illustrate the latter, two trials with a frozen-noise signal are shown in black and blue, respectively). By contrast, with a UD background (Fig. 2*F*), no spiking occurs during the down states, but during the up states the population activity tracks the signal reliably.

We would like to stress that the improved transmission characteristics are simply due to a higher firing rate of the readout population. While in the AI case background spikes occur too sparsely to push readout neurons across threshold, redistributing them into up states allows the readout population to fire and, thus, transmit information about the signal at least in a fraction of the time.

### Quantifying information transmission

To quantify the effect of a UD background, we repeat the procedure for many trials (with different realizations of signal and UD switching) and calculate the spectral coherence between signal and readout activity. The coherence function,

(23) $$C_{sa}(f)=\frac{|S_{sa}(f){|}^{2}}{S_{aa}(f)S_{ss}(f)},$$

attains values between zero and one and indicates how well each frequency component of the signal can be linearly reconstructed from the output. It is calculated from the cross-spectrum, *S_sa_*(*f*), between signal and readout activity and the power spectra, *S_aa_*(*f*) and *S_ss_*(*f*), of activity and signal, respectively.

For an AI background, theoretical expressions for these quantities are known (Richardson and Swarbrick, 2010; Droste and Lindner, 2017a); for a UD background, we have derived a novel approximation (see Materials and Methods). We plot simulation results and theory in Figure 2*G*. It can be seen that the AI coherence is low-pass, which is a known result for integrate-and-fire neurons (Vilela and Lindner, 2009). By contrast, the UD coherence is bandpass, almost vanishing at low frequencies but extending to higher frequencies. This is similar to single neurons subject to a broadband stimulus and a slow background noise (Lindner, 2016) and can be understood by considering that the slow two-state switching hampers signal transmission most strongly at low frequencies: looking only at the readout activity, it becomes difficult to know whether slow fluctuations are actually a part of the signal or a manifestation of the UD switching. Remarkably, the reduction at low frequencies can be overcompensated in the higher frequency range, such that the overall information transmission for the UD case can be higher than in the AI case. The overall information transmission is quantified as follows. As the signal is Gaussian, one may use the coherence to obtain a rigorous lower bound for the mutual information rate (Gabbiani, 1996),

(24) $$R_{\text{info}}^{}=-\int_{0}^{f_{\text{C}}}df\;\log_{2}[1-C_{sa}(f)].$$

In Figure 2*H*, we show $R_{\text{info}}^{}$ for the two scenarios as the mean background rate ${\bar{r}}_{\text{B}}$ is varied. At the intermediate background rate used so far (Fig. 2*H*, dashed line), the information rate is higher for a UD background, which is in line with what we found above. We have argued that this is a firing-rate effect; indeed, with a UD background, the readout population starts to fire at lower background rates (Fig. 2*H*, bottom panel). Notably, at higher background rates, information flow is larger with an AI background.

### Effect of up/down duration and temporal structure of the signal

In experiments, mean up/down state durations ranging from 100 milliseconds to several seconds have been observed (Steriade et al., 1993; Cowan and Wilson, 1994; Stern et al., 1997). Here, we investigate whether the benefits of a UD background occur robustly over such a wide range. Furthermore, we explore the dependence of information transmission on the times spent in up and down states and their interplay with the time scales of the signal.

**Figure 3.**
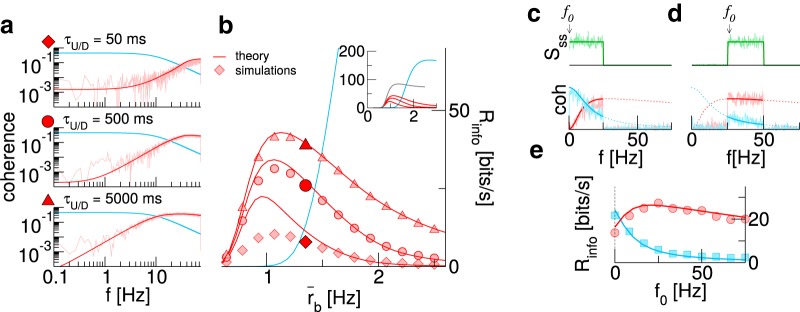
Effects of up/down duration and temporal structure of the signal. ***A***, Coherence for AI background (blue) and UD background at three different values for the mean duration $\tau_{\text{U/D}}$. Here, ${\bar{r}}_{\text{B}}=1.35$ Hz. ***B***, Mutual information rates as a function of the background rate for the cases shown in ***A***. The inset shows the theoretical curves over a wider range; the gray line marks what could optimally be reached by infinitely slow switching. ***C***, ***D***, Signal power spectra and coherence for a lower cutoff *f*_0_ of 0 Hz and 25 Hz, respectively. ***E***, Mutual information rates as a function of *f*_0_. The dashed line marks cutoff frequency used in ***A–C***. In ***A–E***, $\tau_{\text{U}}=333$ ms, $\tau_{\text{D}}=200$ ms, Δf=25 Hz.

The coherence functions shown in Figure 3*A* demonstrate that the benefits of a UD background for signal transmission are robust with respect to the mean residence times. Note that the background firing rates in the two states as well as the mean background rate remain unchanged. Generally, the slower the switching is, the higher is the overall information rate, going along with a more and more pronounced reduction of coherence at low frequencies. Both can be traced back to the temporal correlations of the UD fluctuations which diminish information transmission in a lower and lower frequency band set by the cutoff frequency $f_{\text{UD}}=(1/\tau_{\text{U}}+1/\tau_{\text{D}})$.

What information rate is observed in a scenario with an arbitrarily slow UD background? In this limit, the information rate as a function of the mean background rate attains a particularly simple form in terms of the rate for the AI case:

(25) $$R_{\text{info}}^{\text{UD},\infty }({\overset{¯}{r}}_{\text{B}})=\frac{\tau_{\text{U}}}{\tau_{\text{U}}+\tau_{\text{D}}}R_{\text{info}}^{\text{AI}}(\frac{\tau_{\text{U}}+\tau_{\text{D}}}{\tau_{\text{U}}}{\overset{¯}{r}}_{\text{B}}).$$

Information is transmitted only during the up states that occur with probability $\tau_{\text{U}}/(\tau_{\text{U}}+\tau_{\text{D}})$, and the information rate in these up states is the same as in the AI regime, albeit with a rescaled firing rate. Equation 25 (Fig. 3*B*, inset, gray line) makes clear that the information rate approaches a finite limit and, moreover, suggests that there is a background rate above which a UD background is no longer beneficial (here at $r_{\text{B}}\geq 1.7$ Hz), irrespective of residence times.

The observation that slow signals are more severely affected by UD switching suggests that shifting the power of the signal toward higher frequencies should make the beneficial effect of a UD background even more pronounced. In Figure 3*C–E*, we compare the transmission of signals with constant power in the range $\left[f_{0},f_{0}+\Delta f\right]$. For the sake of illustration, we use here a reduced signal bandwidth Δ*f* = 25 Hz. For *f*_0_ = 0, this choice yields slightly better information transmission with an AI background (Fig. 3*C*,*E*). Increasing *f*_0_ changes this drastically (Fig. 3*D*,*E*). In particular, we observe an optimal lower cutoff frequency, which, for the parameters chosen here, is close to 25 Hz.

### A UD background is also advantageous in a more realistic recurrent network

Up to here, we have made some simplifying assumptions that allowed us to demonstrate and investigate the beneficial effect of a UD background in a tractable setting. Below, we make the setup more realistic in three aspects and show that the observed effect is robust.

**Figure 4.**
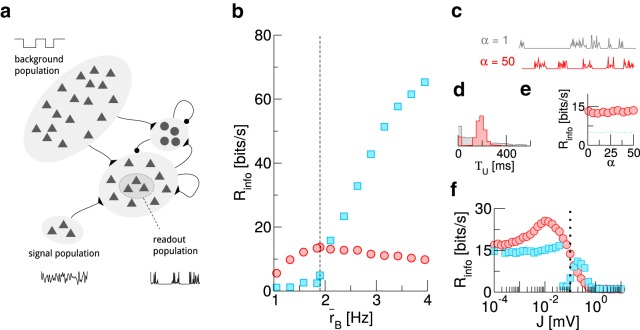
The beneficial effect of a UD background persists in a more realistic setup. ***A***, Sketch of the modified setup. ***B***, Mutual information rate for different mean background rates ${\bar{r}}_{\text{B}}$. The dashed line marks the value used in ***C–F***. ***C***, Sample traces of the readout population activity for irregular (gray, α=1) and rather regular (red, α=50) UD switching. ***D***, Histogram for the duration of up states in the readout population. To obtain this, population activity is binned (width Δ*T* = 10 ms); *n* consecutive bins in each of which at least one readout neuron spikes then define a readout up state of length *T*_U_ = *n*Δ*T*. ***E***, Effect of changing the regularity of UD switching on the mutual information rate. ***F***, Effect of changing the recurrent synaptic weights. The dotted line marks the value used in ***B–E***. In all panels, $\tau_{\text{U/D}}=200$ ms.

The readout population so far consisted of uncoupled neurons that received only excitatory input. To relax this, we now consider a readout population (*N* = 1000) that is a subset of a recurrent network, consisting of 10,000 excitatory and 2500 inhibitory neurons (Fig. 4*A*). The network follows the classical setup by Brunel (2000; for details, see Materials and Methods).

In the previous sections, the durations of UD states were exponentially distributed. While broad distributions have been reported (Stern et al., 1997; Johnson et al., 2010), the statistics seem to be highly dependent on brain state/anesthesia (Chauvette et al., 2011) and can also be rather regular (Steriade et al., 1993). To control switching regularity, we now draw the times spent in UD states from a gamma-distribution, characterized by a shape parameter α. With increasing α, switching becomes more regular, while α=1 recovers the case of exponentially distributed times.

The third change in the setup concerns the signal, which previously entered the neuronal dynamics directly as a current modulation. Now, we introduce a signal population of *N_s_* = 1000 Poisson neurons which encode the signal in their time-dependent firing rate.

As shown in Figure 4*B*, a UD background can still be beneficial for information transmission when background rates are low, even in this more realistic setup.

Changing the regularity of the switching between up and down states in the background has a large effect on the regularity of UD switching in the population activity, as evident from sample traces of *a*(*t*) (Fig. 4*C*) and the histogram of up-state duration (Fig. 4*D*; an up state in the readout population is here defined as a sequence of time bins of Δ*t* = 10 ms in which *a*(*t*) > 0 Hz). In particular, the distribution of times the readout population spends in an up state closely follows that of the background population. Nevertheless, the effect of changing the regularity of the switching on the mutual information rate (and the beneficial nature of the UD background in this context) is negligible (Fig. 4*E*).

Changing the weights of recurrent connections in the network, *J* (Fig. 4*F*) has a strong effect on signal transmission properties, both for UD and AI backgrounds. Here, it is plausible that $R_{\text{info}}^{}$ goes to zero in the limit of a very strongly coupled network, in which intrinsic dynamics dominate the population activity. Remarkably, the information rate with UD background shows a pronounced maximum at intermediate synaptic strength. This is indicative of the rich dynamics that recurrent networks exhibit and constitutes an interesting subject for future work.

### Effect of the signal on the UD switching

A number of experimental studies have described an effect of the sensory signal on the UD switching (Shu et al., 2003; Reig and Sanchez-Vives, 2007). In our original setup, the UD switching happens in a background population that is unaffected by the signal. It is, however, interesting how such signal-dependence of the UD transitions influences their benefit for information transmission. While for a thorough study, one would need to explicitly model the mechanism underlying the UD transitions to allow the signal to influence them, we pursue a more phenomenological approach here. We assume that the background population also receives the signal and that it enters as a modulation of the switching rates $k_{\pm }$ (from up to down and vice versa), which now become time dependent,

(26) $$k_{+}(t)={\overset{¯}{k}}_{+}[1-\epsilon_{k}s(t)],$$

(27) $$k_{-}(t)={\overset{¯}{k}}_{-}[1+\epsilon_{k}s(t)],$$

where the ${\overset{¯}{k}}_{\pm }$ now stand for the original (constant) rates and $\epsilon_{k}$ quantifies how strongly UD transitions are influenced by the signal *s*(*t*). We assume that a positive signal tends to keep the population in an up state (thus reducing the rate of leaving it), while it makes a down state shorter; hence the different signs in Equations 26, 27.

**Figure 5.**
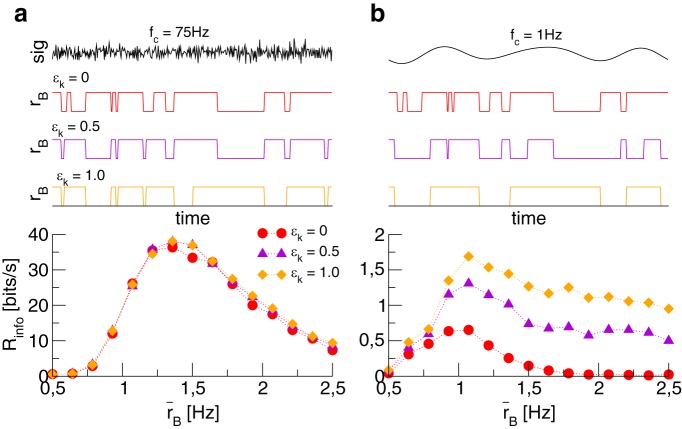
A simple model for the effect of the signal on the UD switching We assume that the transition rates between UD states in the background are modulated by the signal. Shown is a realization of the signal and the corresponding background rate for three values of the modulation strength $\epsilon_{k}$, as well as the mutual information rate over the mean background rate (obtained in simulations) for these $\epsilon_{k}$. ***A***, Parameters as in Figure 2. ***B***, A much slower signal with *f*_C_ = 1 Hz. Note the different *y*-axis scaling in the mutual information plots.

In Figure 5, we plot the mutual information rate with an UD background for different values of $\epsilon_{k}$. We also show sample traces of the signal and the respective time-dependent firing rates of the background population (to make these traces comparable, the same sequences of random numbers have been used). The signal-dependence of the UD switching has only a small effect on the mutual information rate, namely a slight overall increase. This is consistent with the fact that more signal power is fed into the system (hence the increase). This power, however, is strongly low-pass filtered by the comparatively slow UD switching so that only information about low frequencies reaches the neurons in the readout population via the background (hence the weakness of the effect). Consequently, we see a much more pronounced effect of the signal influencing the U/D transitions, if the signal power is restricted to a very low frequency band (Fig. 5*B*). Qualitatively, in all cases the beneficial effect of UD switching persists.

### Traveling waves of up states allow continuous signal transmission

**Figure 6.**
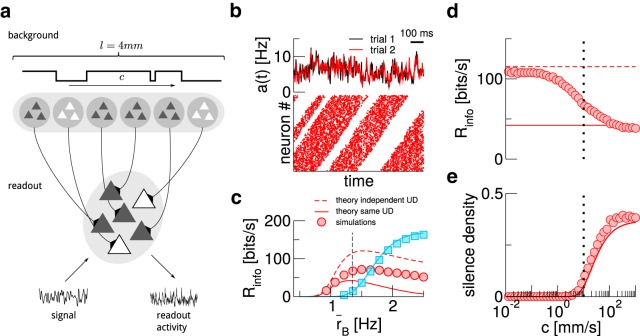
Traveling up states allow continuous signal transmission ***A***, Sketch of the setup. ***B***, Activity and spike raster of the readout population (neurons ordered by the position of their background population). ***C***, Mutual information rates for different background rates. Red solid and dashed lines correspond to the theoretical limits c→∞ and c→0, respectively; blue line is theory for AI background. The black dashed line marks the background rate used in the other panels. ***D***, Mutual information rates for varying wave speed *c*. The dotted line marks the wave speed used in ***B***, ***C***. ***E***, Silence density, i.e., fraction of time bins (length Δ*T* = 4 ms) in which the population activity is zero. Where nothing else is indicated, the wave speed is *c* = 10 mm/s and the mean background rate is ${\bar{r}}_{\text{B}}=1.35$ Hz. In ***C–E***, symbols represent simulation results, while lines are theory.

Experiments have shown that the switching between up and down states is not always simultaneous for distinct neurons in a cortical region, but instead often occurs in the form of traveling waves (Amzica and Steriade, 1995; Sanchez-Vives and McCormick, 2000; Petersen et al., 2003; Luczak et al., 2007). This motivates us to extend the setup from Figure 2 to incorporate traveling UD states in a simple way. We consider the neurons of the background population to be distributed uniformly along one space dimension (length *l* = 4 mm). Note that the spatial extent of the readout population will in general not correspond to that of the background but can be much smaller, implying a strong overlap in receptive fields of the read-out neurons, which motivates the global nature of our input signal. As before, each readout neuron receives Poisson input with a two-state rate, but now the switching in this rate does not happen simultaneously across neurons but propagates with a constant velocity *c* across the background population (Fig. 6*A*, sketch). As *c* and $\tau_{\text{U/D}}$ are the quantities that are typically measured in experiments, we use them as key parameters of the system. This means in particular that the average spatial extent of an up/down state, $\lambda_{\text{U/D}}=c\tau_{\text{U/D}}$, varies if we vary *c* or $\tau_{\text{U/D}}$.

In the context of information transmission, traveling UD states can remedy an important conceptual problem. As discussed above, information transmission with a UD background benefits if the mean duration of up and down states is long, i.e., if the switching between the states is as slow as possible. However, a high average rate of information transmission (as quantified by $R_{\text{info}}^{}$) may be of little use to an animal if transient stimuli are missed because they fall into a down-state interval. If UD states do not occur simultaneously but instead propagate across cortex, however, this problem can be circumvented, as has been previously suggested in the context of periodic waves (Ermentrout and Kleinfeld, 2001): for appropriately chosen parameters (such that $\lambda_{\text{D}}<l$), there should almost always be a fraction of readout neurons that receive an up-state background and transmit the signal. Indeed, for a physiologically realistic propagation speed *c* = 10 mm/s ($\lambda_{\text{D}}=2$ mm), continuous tracking of the signal works surprisingly well (Fig. 6*B*).

In Figure 6*C*, we plot the mutual information rate for different background rates. We also plot two limit cases: for waves that propagate infinitely fast (c→∞, implying λ→∞; solid red line), state changes spread instantaneously through the population and one recovers the case where UD switching occurs simultaneously (Fig. 2), while for infinitely slow propagation (c→0,λ→0; dashed red line), each readout neuron receives an independent two-state process. The latter limit yields higher information transmission rates because compared to the case of instantaneous propagation, in which neurons are synchronized by the simultaneous UD states, they become decorrelated here. Varying *c* interpolates between these limits (Fig. 6*D*): the information rate with a traveling UD background is always larger than with simultaneous switching.

To further quantify how traveling UD states enable continuous signal transmission, we measure the silence density (Mochol et al., 2015), defined as the fraction of time bins in which the population activity is zero (shown in Fig. 6*E* along with a theoretical prediction, see Materials and Methods). For a propagation velocity c≲3 mm/s, this is practically zero, and already for *c* = 10 mm/s (dashed line), it has decayed to ≈10% of the value with instantaneous switching.

## Discussion

We have studied theoretically how ongoing background activity affects the information transmitted about a signal. We find that signal transmission can benefit from a background that undergoes transitions between up and down states, compared to an AI background with the same mean firing rate. This is a surprising result, bearing in mind that the stochastic UD switching, which is unrelated to the signal, dominates the temporal structure of the readout activity. UD switching is favorable at low background rates, where an AI background elicits only weak spiking in the readout population. Here, up states allow at least brief periods with elevated firing rates, during which information rates are high enough to overcompensate for silent periods and UD-induced correlations. Taking into account that up states propagate as traveling waves makes the gain in information compared to the AI case even stronger. It also enables continuous signal transmission, addressing the concern that short transient signals might be missed during silent periods.

### Generality and robustness

For our comparison of AI and UD regimes, we have focused on information transmission as a measure, a paradigm also used in experimental studies. This raises the question whether the beneficial effect of a UD background that we observe carries over to more complex computational tasks, in which information is not merely forwarded, but processed. Indeed, it seems plausible that a UD background also benefits information processing, simply because it is difficult to envision how the very low readout firing rates in the AI case could possibly be a preferable choice. An interesting quantitative approach to this question could be a comparison of UD and AI backgrounds in a network that is able to produce a high-dimensional representation of a signal, which is believed to facilitate computation by downstream neurons (Buonomano and Maass, 2009; Ostojic, 2014).

A deliberate limitation of our model is the clear separation between the UD background and the readout population. An alternative would have been to equip the readout population with a mechanism to generate UD switching dynamically (Contreras et al., 1996; Sanchez-Vives and McCormick, 2000). As it would have become difficult to disentangle the effects of specific mechanisms from general consequences of UD transitions, we chose not to include endogenous generation of UD switching in the readout population.

Other simplifications in our model have only little effect. As we have demonstrated, the beneficial effect of a UD background persists when the readout population contains recurrent connections (including local inhibition), when the statistics of the UD switching are changed, or when the signal is encoded in input spikes, rather than entering as a current. We also have checked that the effect does not hinge on the exact value of the ratio of mean up and down durations within a physiologic range (we used $\tau_{\text{U}}/\tau_{\text{D}}=1$ in Fig. 3 and $\tau_{\text{D}}/\tau_{\text{D}}\approx 1.7$ everywhere else). Even if we permit the signal to influence the switching rates of the UD background, this changes the information rates only slightly. The mechanism is robust because it is simple. This suggests that the effect would also persist if we included more realistic neuron models (such as the aEIF model; Brette and Gerstner, 2005), heterogeneity between neurons (Harrison et al., 2015), and recurrent connections (Litwin-Kumar and Doiron, 2012), or an overlap of background spike trains (de La Rocha et al., 2007), to name but a few possibilities.

### Transient signals and silent periods

Down states in the background cause collective silent periods in the readout population. This poses a problem for signal transmission, because short but potentially important transient signals might be missed completely. There are at least two possible mechanisms to mitigate this. First, transient stimuli can influence the UD switching (Shu et al., 2003; Reig and Sanchez-Vives, 2007). This suggests that transient inputs could make themselves heard by causing an up state, while spontaneously occurring up states probe the input for more stationary features, in line with Luczak et al.(2013). Second, UD switching does in general not happen simultaneously across a patch of cortex; instead, up-state activity propagates as a traveling wave (Amzica and Steriade, 1995; Sanchez-Vives and McCormick, 2000; Petersen et al., 2003; Luczak et al., 2007). As we have demonstrated, this enables nearly continuous signal transmission for physiologically realistic parameters. Note that the idea that traveling waves ensure “that only part of the sensory field is rendered unresponsive” has already been brought up in the context of oscillatory waves (Ermentrout and Kleinfeld, 2001).

### Relation to previous studies

Most theoretical studies concerned with UD states have primarily focused on their dynamical generation (Latham et al., 2000; Compte et al., 2003; Holcman and Tsodyks, 2006; Destexhe, 2009; Mochol et al., 2015). Stimulus transmission has received less attention (Curto et al., 2009; Reig et al., 2015), and we are not aware of previous theoretical studies that consider the information transmitted about time-dependent stimuli. Methodologically, we have built on analytical approaches used to describe signal transmission with AI backgrounds (Lindner and Schimansky-Geier, 2001; Fourcaud and Brunel, 2002; Richardson and Swarbrick, 2010). We have extended these approaches to a UD background, modeling the background rate as a two-state process. Comparison with simulations shows that our approximation (mainly based on the assumption of sufficiently long down states) works remarkably well for physiologically realistic parameter values.

Recently, several experimental studies have compared stimulus transmission during AI (desynchronized) and UD (synchronized) regimes (Goard and Dan, 2009; Marguet and Harris, 2011; Luczak et al., 2013; Zagha et al., 2013; Pachitariu et al., 2015). In these studies, multi-unit activity from a sensory cortex was measured during anesthesia or quiet wakefulness where UD switching could be observed. Transitions from UD to AI regimes were either allowed to happen spontaneously or induced. The response to a sensory stimulus was then compared across the two conditions. All studies found that the transmission of the stimulus was strongly improved in AI regimes; during UD regimes, information was also transmitted, but at a lower rate. In the AI case, correlations among simultaneously recorded neurons were reduced while correlations across trials (with a frozen stimulus) were increased (Goard and Dan, 2009; Pachitariu et al., 2015). Similarly, spiking activity could be better predicted from the stimulus in the AI regime, while the local field potential was a better predictor in the UD case (Marguet and Harris, 2011).

**Figure 7.**
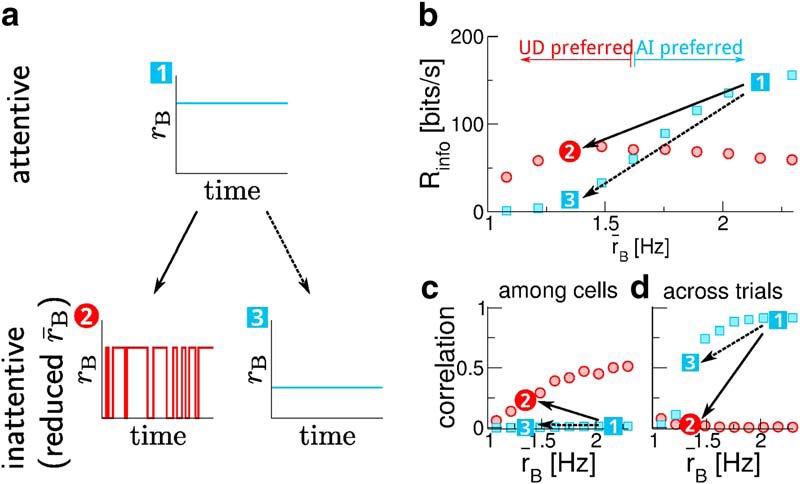
What do our results say about the comparison of different brain states? ***A***, We consider a switch from an attentive state (1) to an inattentive state at a lower mean background firing rate ${\bar{r}}_{\text{B}}$. The background rate can either be lowered by introducing pauses (going to the UD regime (2)) or in a uniform manner (going to the AI regime (3)). ***B***, Switching from (1) to (2) maintains higher information rates than switching to (3); we propose that this is why (2), not (3), is observed. In both cases, the overall information transmission is reduced. ***C***, This goes along with an increase in noise correlations (correlations among neurons within one trial; shown is the mean Pearson correlation coefficient of spike counts in 100-ms time bins; see Materials and Methods) and (***D***) a decrease in signal correlations (correlations in the binned population activity across trials; see Materials and Methods). All parameters are chosen like in Figure 6; in ***B–D***, symbols represent simulation results.

It is important to note that the comparison conducted in the cited experimental studies is not equivalent to the comparison in this work: while we contrast the two regimes at a fixed background rate, the experimental studies compare different brain states, which are likely to involve different background rates (Poulet et al., 2012). In Figure 7, we illustrate what such a comparison between brain states (characterized by different background rates) means in our model. We consider a transition from an “attentive state” (marked by 1 in Fig. 7) to an “inattentive state” at a reduced rate. A motivating question of our work was why the rate should be lowered via down states (1 → 2 in Fig. 7) instead of in a uniform manner (1 → 3 in Fig. 7). Our results suggest that the regime 2 rather than 3 is observed because it is advantageous for information transmission (Fig. 7*B*). This comparison, the UD regime 2 versus the AI regime 3, is the one we have focused on in this work, whereas the comparison in the cited experimental studies is probably closer to one between the AI regime 1 and the UD regime 2. Indeed, for this second comparison, our results are consistent with the experimental observations: more information is transmitted in the AI case 1 (Fig. 7*B*), which shows less correlations among simultaneously recorded neurons (noise correlations, Fig. 7*C*), but higher correlations across trials (signal correlations, Fig. 7*D*).

Besides the UD transitions (slow oscillations) there are a number of other rhythms present in brain activity such as gamma oscillations, thalamic spindles and hippocampal sharp waves and ripples (Steriade, 2006). Spindles and sharp waves supposedly play a major role in the long-term consolidation of memories, e.g., the migration of mnemonic representations from hippocampus to neocortical areas (Buzsáki, 1996), and may be controlled by UD transitions to facilitate the consolidation process (Staresina et al., 2015). Gamma oscillations may be used for multiplexing information transfer efficiently (for an example from the visual system, see Koepsell et al., 2010). More generally, brain oscillations may mediate components of higher-level sensory perception, including perhaps human consciousness (Hopfield and Brody, 2001; Singer, 2001). In contrast to these and other specific roles of brain rhythms in neural information transmission, the beneficial effect of a UD background under the constraint of a low overall firing rate seems to be rather generic and thus potentially relevant for the information transfer in many brain areas.

### Functional consequences

Our finding that UD switching can improve information transmission at lower background rates leads us to hypothesize that such a regime could represent a compromise between reducing metabolic cost (allowing cellular regeneration; Vyazovskiy and Harris, 2013) and maintaining information transmission capabilities. Similar to the suggestion that (periodic) traveling waves could be “means to scan the incoming sensory stream for novel features” (Ermentrout and Kleinfeld, 2001) or that up states represent the “sporadic opening of a ‘gate’” for the transmission of sensory signals (Luczak et al., 2013), we propose that below a certain level of activity, UD states emerge because they allow for a maximized information transmission under such constraints.

## Synthesis

Reviewing Editor: Tatyana Sharpee, The Salk Institute for Biological Studies Decisions are customarily a result of the Reviewing Editor and the peer reviewers coming together and discussing their recommendations until a consensus is reached. When revisions are invited, a fact-based synthesis statement explaining their decision and outlining what is needed to prepare a revision will be listed below. The following reviewer(s) agreed to reveal their identity: Brent Doiron

The manuscript shows how brief epochs of high firing rates during up-down dynamics can offer improved coding over the low firing rates during asynchronous firing. Please discuss the contribution of brain rhythms other than slow waves that contribute to information transfer (spindles, hippocampal sharp wave, ripples). Please include references to papers on the role of sleep in memory consolidation.

Reviewer 1:

The authors also refer to quiet wakefulness as an example of state with UD. While occasional slow waves have been reported in awake, particularly after sleep deprivation, the quiet wakefulness is dominated by alpha (and some related) activities which are very different from SO.

Although the authors are upfront by stating that they compared two regimes (i.e., asynchronous irregular (AI) and (UD) at a fixed background rate, the motivation for having a uniform transition from AI to UD is not clear to me. Is there any evidence for switching between two states with same firing rates?

From Figure 2b it seems that firing rate is the same during Up states but the UD duty-cycle (ratio of up state to a cycle period) changes over time (probably because of exponential distributions). For such a waveform with no alteration in average firing rate, a constant ratio of up state to down state is required. Please clarify how you selected this ratio and confirm if this ratio was always the same throughout the paper. I wonder if changing this ratio affects the resulted mutual information rates.

Regardless of coherence computations, it is not conceptually clear why having pauses in background activity changes coherence (and information transfer rate) function from low-pass (high-pass) to band-pass. Adding an explanation would be helpful. Authors also concluded more information transfer was resulted by UD regime for lower background rates. Please provide a range. Does this range have overlap with UD state in the brain?

It seems that the statement of “the slower switching, the higher is the overall information rate” may not be a proper conclusion. Because, increasing an UD cycle period would probably also increase the up-state firing rate to keep the mean background rate unchanged. I suggest testing up/down duration effect in case of same up-state firing rates. Please also clarify what infinitely slow switching actually mean.

Minor comments

- Define c in equation (7). Is xn in equation (7) the one used in equation (3)?

- The exponent sign in Fourier transform formulation (in equation 8 and one before equation 10) should be negative.

- No statistical tests were used for performed comparisons (for example, comparisons in figures 2h, 4e, and 5).

Reviewer 2:

Overall, I agree with reviewer 1 that the simplifications place severe limitations on the overall impact of the work. Nonetheless, it is an important start that can be improved upon in subsequent research. I particularly like the interpretation of why “inattentive” states typically show up-down dynamics compared to low rate asynchrony, and how it also explains the higher degree of correlations during attention. A recent study that links up-down dynamics and attention that is relevant is:

Selective modulation of cortical state during spatial attention. Engel, Tatiana A and Steinmetz, Nicholas A and Gieselmann, Marc A and Thiele, Alexander and Moore, Tirin and Boahen, Kwabena, Science:354, 1140-1144, 2016.
